# Potential roles of IL-38, among other inflammation-related biomarkers, in predicting post-percutaneous coronary intervention cardiovascular events

**DOI:** 10.3389/fcvm.2024.1426939

**Published:** 2024-08-02

**Authors:** Lu Kou, Ning Yang, Bo Dong, Qin Qin

**Affiliations:** Department of Cardiology, Tianjin Chest Hospital, Tianjin, China

**Keywords:** post-PCI, MACE occurrence prediction, multivariate logistic regression analysis, serum IL-38, serum hs-CRP, serum HbA1c

## Abstract

Percutaneous coronary intervention (PCI), as a relatively rapid and effective minimally invasive treatment for coronary heart disease (CHD), can effectively relieve coronary artery stenosis and restore myocardial perfusion. However, the occurrence of major adverse cardiovascular events (MACE) is a significant challenge for post PCI care. To better understand risk/benefit indicators and provide post PCI MACE prediction, 408 patients with CHD who had undergone PCI treatment from 2018 to 2021 in Tianjin Chest hospital were retrospectively studied for their clinical characteristics in relation with the MACE occurrence during a 12-month follow-up. In the study, 194 patients had MACE and 214 patients remained MACE-free. Using uni- and multivariate regression analyses, we have shown that smoking history, elevated serum C-reactive protein levels (hs-CRP), and high haemoglobin levels A1c (HbA1c) are all independent risk factors for MACE after PCI. Furthermore, we have discovered that the serum level of IL-38, one of the latest members identified in the IL-1 cytokine family, is another predictive factor and is reversely related to the occurrence of MACE. The serum level of IL-38 alone is capable of predicting non-MACE occurrence in subcategorized patients with abnormal levels of hs-CRP and/or HbA1c.

## Introduction

Coronary heart disease (CHD) is a condition caused by myocardial hypoxia, ischaemia, or necrosis due to vascular lumen stenosis or obstruction, and has a high mortality rate and a disability rate (1). Metabolic diseases such as type 2 diabetes mellitus (T2DM) are a common concomitant of coronary heart disease and an independent risk factor for its occurrence. In 2015, there were 415 million adults with diabetes worldwide, and 75% of the people with T2DM died of cardiovascular disease (3, 4). The combination of metabolic disorders and CHD will not only dramatically increase the treatment difficulty, but also lead to the deterioration of CHD, and even acute myocardial infarction, threatening the life and health of patients (2). Percutaneous coronary intervention (PCI), as a relatively rapid and effective minimally invasive treatment for CHD, can effectively relieve coronary artery stenosis and restore myocardial perfusion (3). However, in patients with CHD complicated with metabolic disorders, the incidence of major adverse cardiovascular events (MACE) after PCI is significantly increased (4). To minimize MACE occurrence in patients with CHD, it is particularly crucial to evaluate the clinical characteristics and the risk/benefit indicators before PCI is performed.

It has been reported that inflammation, indicated by C-reactive protein (hs-CRP), and hyperglycaemia, indicated by haemoglobin A1c (HbA1c), jointly contribute to the cardiovascular risk of patients with metabolic disease complications. Patients with both high hs-CRP and HbA1c (>0.44 mg/dl and >6.2%, respectively) are at particularly high risk for poor cardiovascular outcomes (5). Therefore, tackling inflammation is a strategy many take to control cardiovascular risk both before and after surgical treatments like PCI.

As one of the major members in inflammation pathway, interleukin (IL)-1 family is involved heavily in regulating immune responses, and many studies and experimental approaches have implicated members of the IL-1 family and their receptors as being significantly associated with cardiovascular diseases (6). IL-38, one of the latest members identified in the IL-1 cytokine family, has been reported to act as an immune modulator, primarily by blocking the release of pro-inflammatory cytokines (7). A meta-analysis of a genome-wide association study (GWAS) found that IL-38 was strongly correlated with CRP levels (8). Recent findings supported the involvement of IL-38 in a variety of conditions, including gestational diabetes mellitus (GDM) and paediatric T2DM (9). Plasma IL-38 was also observed to be higher in T2DM patients and positively related to HbA1c (10). It was reported that a relative deficiency of IL-38 is associated with increased systemic inflammation in ageing, cardiovascular, and metabolic diseases, and is consistent with IL-38 as an anti-inflammatory cytokine (11). Therefore, it is reasonable to assume that IL-38 level has impact on cardiovascular risk, including MACE occurrence post treatment.

To further explore how IL-38 is correlated with the appearance of MACE after PCI, this study retrospectively analyzed the clinical characteristics of patients with CHD who were treated in recent years, and identified the risk factors for MACE after PCI to provide reference for future treatment and prognosis.

## Methods

### Patient cohort and data collection

**Figure 1:**
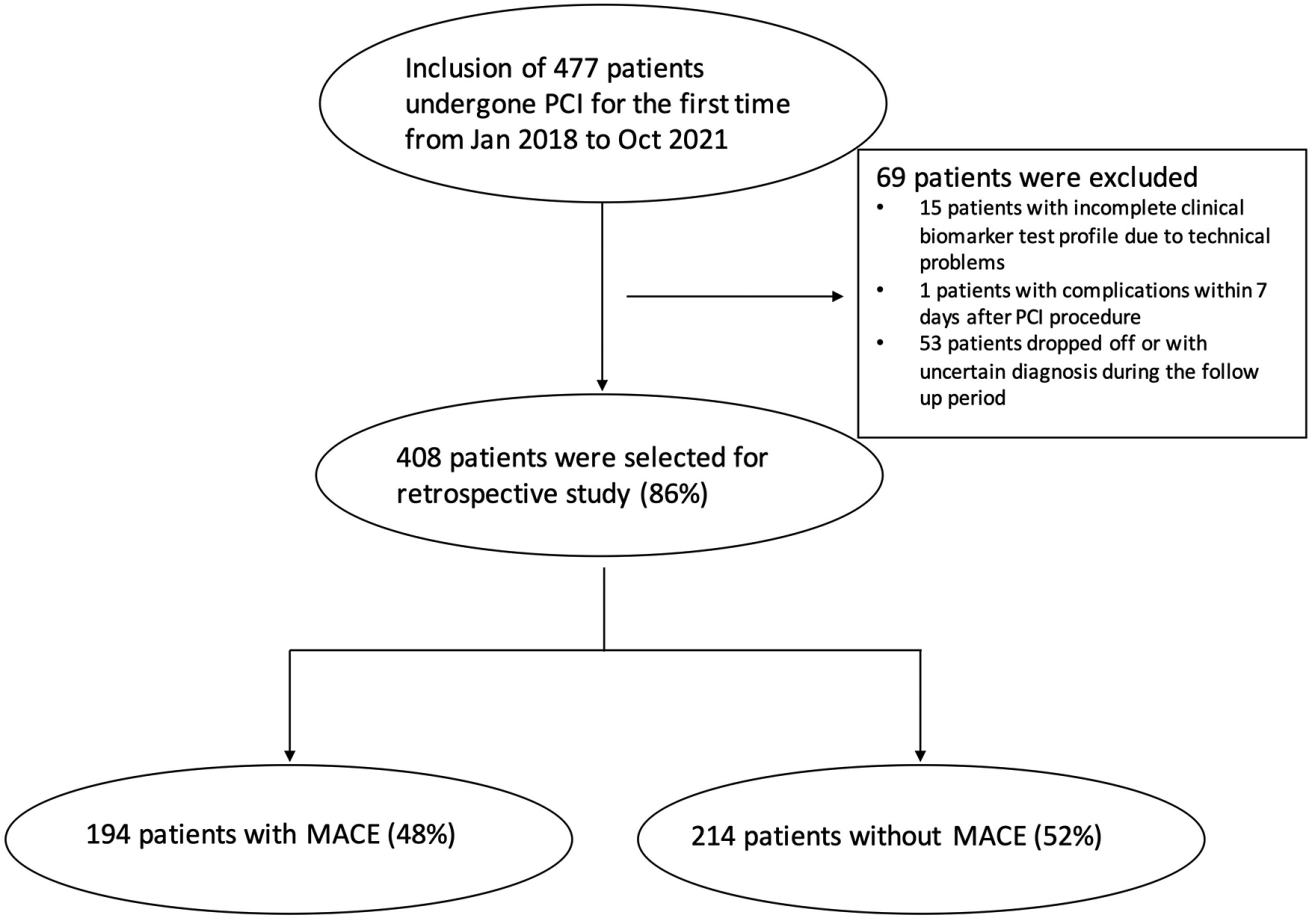
Flow chart of the patient sample size and inclusion/exclusion criteria.

A total of 477 patients who underwent PCI for the first time at the Tianjin Chest Hospital Cardiovascular Disease Department between January 2018 and October 2021 were reviewed in the study. After careful selection, 69 patients were excluded. Among them 15 patients had incomplete clinical biomarker test profiles due to technical problems; 1 patient developed MACE within 7 days after PCI procedure, indicating possible procedure-induced complications; and 53 patients dropped off or were with uncertain diagnosis during the follow-up period. The remaining 408 (86%) patients then formed the cohort of this study, as shown in Figure 1. The study protocol was approved by the Ethics Committee of the Tianjin Chest Hospital. The human samples used in this study were acquired from a by-product of routine care or industry. Written informed consent for participation was not required from the participants or the participants’ legal guardians/next of kin in accordance with the national legislation and institutional requirements.

The general clinical variables of all patients were collected at the time of admission, including gender, age, blood pressure, CHD course, concomitant underlying diseases, and history of smoking. The occurrence of MACE was recorded during the 12-month follow-up period.

### Biochemical indicators

Peripheral blood was collected from all patients after 12 h of fasting. The level of haemoglobin A1c (HbAlc) was determined by the glycated haemoglobin metre (Bio-Rad, Berkeley, CA, USA). OLYMPUS AU640 automatic biochemical analyzer (Beckman, Brea, CA, USA) and supporting reagents were used to detect hs-CRP, serum triglycerides (TG), total cholesterol (TC), high-density lipoprotein cholesterol (HDL-C), and low-density lipoprotein cholesterol (LDL-C) levels, and the operation was carried out in accordance with the standard operating procedures for instruments. The levels of plasma IL-38 (Adipogen, San Diego, CA, USA) were measured by an enzyme-linked immunosorbent assay (ELISA), following the manufacturer's instructions. Briefly, 100 µl of serum sample from each patient was used with IL-38 (human) Matched Pair Detection Set (Adipogen, AG-46B-0007-KI01) to develop sandwich ELISA in duplicates. The average of the readings was then used to calculate IL-38 concentrations against the pre-generated standard curve. The minimal detectable concentration was 40 pg/ml. The ELISA intra-assay and inter-assay coefficients of variation were <5% and <10%, respectively.

### Statistical processing

SPSS (26.0) software was applied to analyze the experimental data in the study. The difference of the discrete variables was expressed as *x* (%) and evaluated using *χ*^2^ test. The difference of the continuous variables was expressed as “$\bar{x}\pm s$” and analyzed using the two-sample *t*-test. Multivariate logistic regression model was used to assess the independent risk factors for MACE. Box plots and receiver operating characteristic curves (ROC) were drawn to test the interactions between serum IL-38 level and relevant risk factors in cohort subgroups. A two-sided *p* <0.05 was considered as a statistically significant difference.

## Results

### Association of occurrence of MACE after PCI and risk factors in patients’ cohort

During the follow-up period, 194 patients with MACE occurrences, representing 48% of the cohort, were classified as the MACE group; while 214 patients who had no adverse cardiovascular events or endpoint events were classified as the non-MACE group (Figure 1). MACE events include unstable angina, congestive heart failure, repeat PCI, non-fatal myocardial infarction, and cardiac death.

Univariate analysis showed that there were significant differences between the MACE and non-MACE groups in terms of smoking history, levels of HbAlc, HDL-C, and hs-CRP (*p* < 0.05), as presented in Table 1.

**Table 1:** Comparison of baseline clinical characteristics and biochemical index levels between the MACE and non-MACE groups.

Index	Cohort (*n* = 408)	MACE (*n* = 194)	Non-MACE (*n* = 214)	*p*-value (*χ*^2^/*t* or *t*-test)
Gender (male/female)	213/195	101/93	112/102	0.955
Age (years)	60.12 ± 8.62	60.42 ± 8.97	59.86 ± 8.31	0.513
Smoking history	180 (44.12)	103 (53.09)	77 (35.98)	0.001
Hypertension	248 (60.78)	119 (61.34)	129 (60.28)	0.827
HbAlc (%)	6.33 ± 1.17	6.89 ± 1.32	5.82 ± 0.71	0.000
TC (mmol/L)	4.98 ± 1.23	5.07 ± 1.33	4.89 ± 1.12	0.146
TG (mmol/L)	1.77 ± 1.06	1.85 ± 0.95	1.69 ± 1.14	0.129
HDL-C (mmol/L)	1.30 ± 0.30	1.25 ± 0.27	1.34 ± 0.31	0.002
LDL-C (mmol/L)	2.96 ± 1.05	3.06 ± 1.12	2.86 ± 0.98	0.053
IL-38 (ng/ml)	6.00 ± 2.26	5.88 ± 1.43	6.11 ± 2.82	0.303
hs-CRP (mg/L)	5.14 ± 2.00	5.81 ± 2.09	4.53 ± 1.71	0.000

Multivariate logistic regression analysis showed the risk of occurrence of MACE after PCI was significantly higher in patients with smoking history, and higher level of HbAlc and hs-CRP, with adjusted odds ratios (95% CI) of 3.55 (2.02–6.26), 4.46 (2.97–6.70), or 1.61 (1.34–1.94), respectively, as presented in Table 2. However, HDL-C level became a non-significant (*p *> 0.05) risk factor after adjustment of its interaction with the other variants. More interestingly, given the potential interaction with other variables, serum IL-38 was included in the logistic regression model (12), and the analysis showed its level reversely correlates to MACE occurrence with OR value of 0.64 (0.55–0.74), appearing to be one significant risk prediction factor for MACE after PCI.

**Table 2:** Association of occurrence of MACE after PCI and risk factors using multivariate logistic regression analysis.

Index	OR (95% CI)	*p*-value
Smoking history	3.55 (2.02–6.26)	<0.001
HbAlc (%)	4.46 (2.97–6.70)	<0.001
hs-CRP (mg/L)	1.61 (1.34–1.94)	<0.001
HDL-C (mmol/L)	0.23 (0.04–1.26)	0.09
IL-38(ng/ml)	0.64 (0.55–0.74)	<0.001

### Serum Il-38 level in subgroups of patients against MACE occurrence

Given the strong link between IL-38 and hs-CRP, as well as IL-38 and HbA1c in CHD risk association, although the previous univariate study did not show direct IL-38–MACE occurrence correlation, the multivariate logistic regression model suggests that IL-38 may contribute to the risk reduction through interaction with other independent risk factors. To further investigate this, serum IL-38 levels were analyzed against MACE occurrence in different subgroups of patients with smoking history, high hs-CRP levels, and high HbA1c levels, respectively.

**Figure 2:**
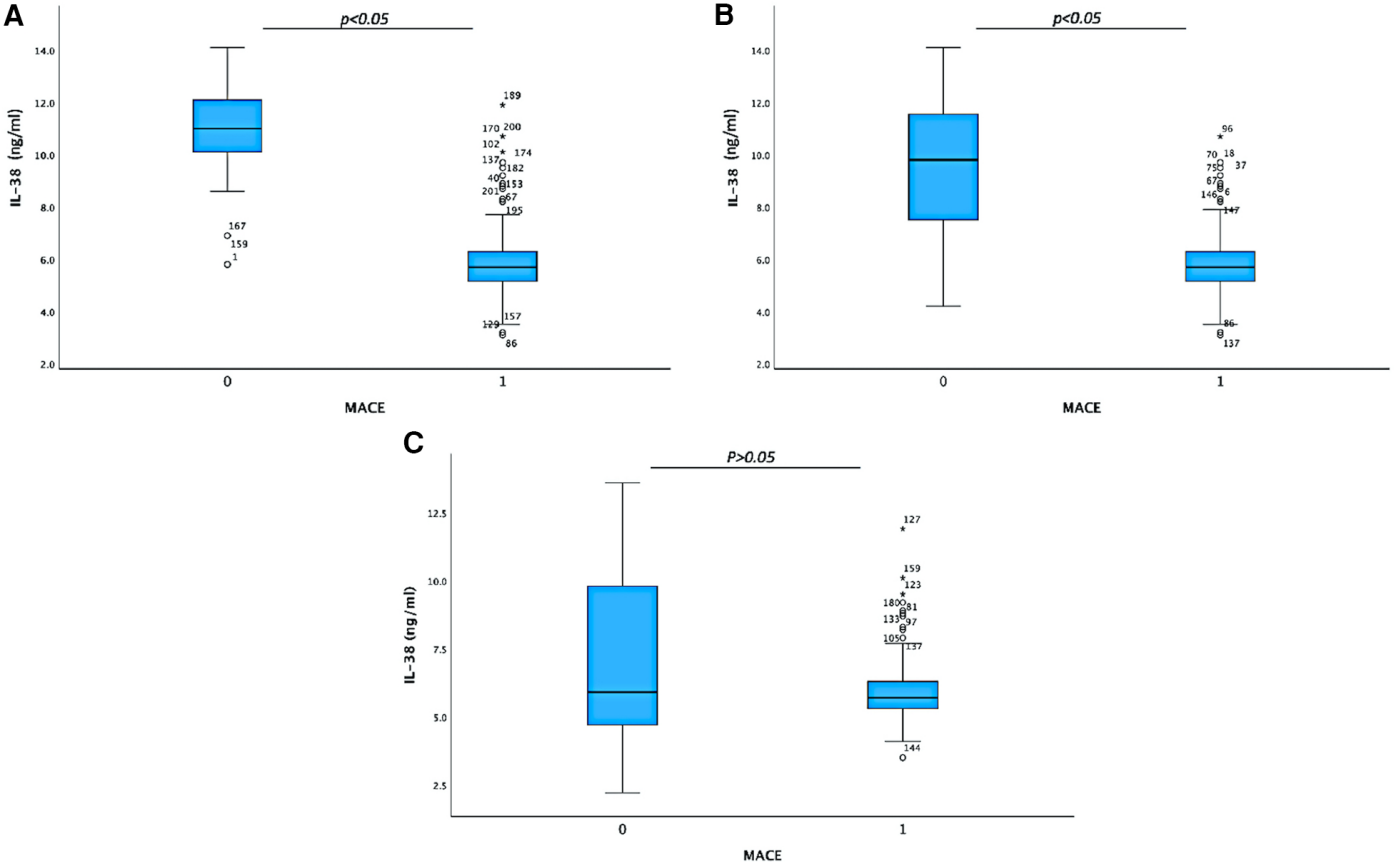
Box plot on serum IL-38 levels in different patient subgroups. (**A**) Serum IL-38 level analyzed between non-MACE (29) and MACE (172) patients in the group with serum hs-CRP higher than 4.4 mg/L, with median IL-38 levels of 11.0 and 5.7 ng/ml, respectively (*p *< 0.05). (**B**) Serum IL-38 level analyzed between non-MACE (47) and MACE (140) patients in the group with serum HbA1c higher than 6.2%, with median IL-38 levels of 9.7 and 5.8 ng/ml, respectively (*p *< 0.05). (**C**) Serum IL-38 level analyzed between non-MACE (72 ) and MACE (112 ) patients in the group with smoking history, with median IL-38 levels of 6.0 and 5.7 ng/ml, respectively (*p *> 0.05).

As shown in Figure 2, we found that in the high hs-CRP subgroup, the serum IL-38 level was significantly lower in the 172 patients with MACE compared with that in the 29 non-MACE patients with its median level of 5.7 and 11.0 ng/ml, respectively (*p* < 0.05) (Figure 2A); likewise in the high HbA1c subgroup, the serum IL-38 level was significantly lower in the 140 patients with MACE compared with that in the 47 non-MACE patients with its median level of 5.8 and 9.7 ng/ml, respectively (*p* < 0.05) (Figure 2B), indicating that IL-38 expression is a reverse indicator for MACE occurrence risk in patient subgroups with higher hs-CRP and/or HbA1c. Conversely, there was no significant difference in IL-38 levels between the 112 MACE and the 72 non-MACE patients in the smoking subgroup, with its median levels of 5.7 and 6.0 ng/ml, respectively (*p *> 0.05), indicating there is no direct interaction between IL-38 and smoking history in post-PCI MACE prediction (Figure 2C).

**Figure 3:**
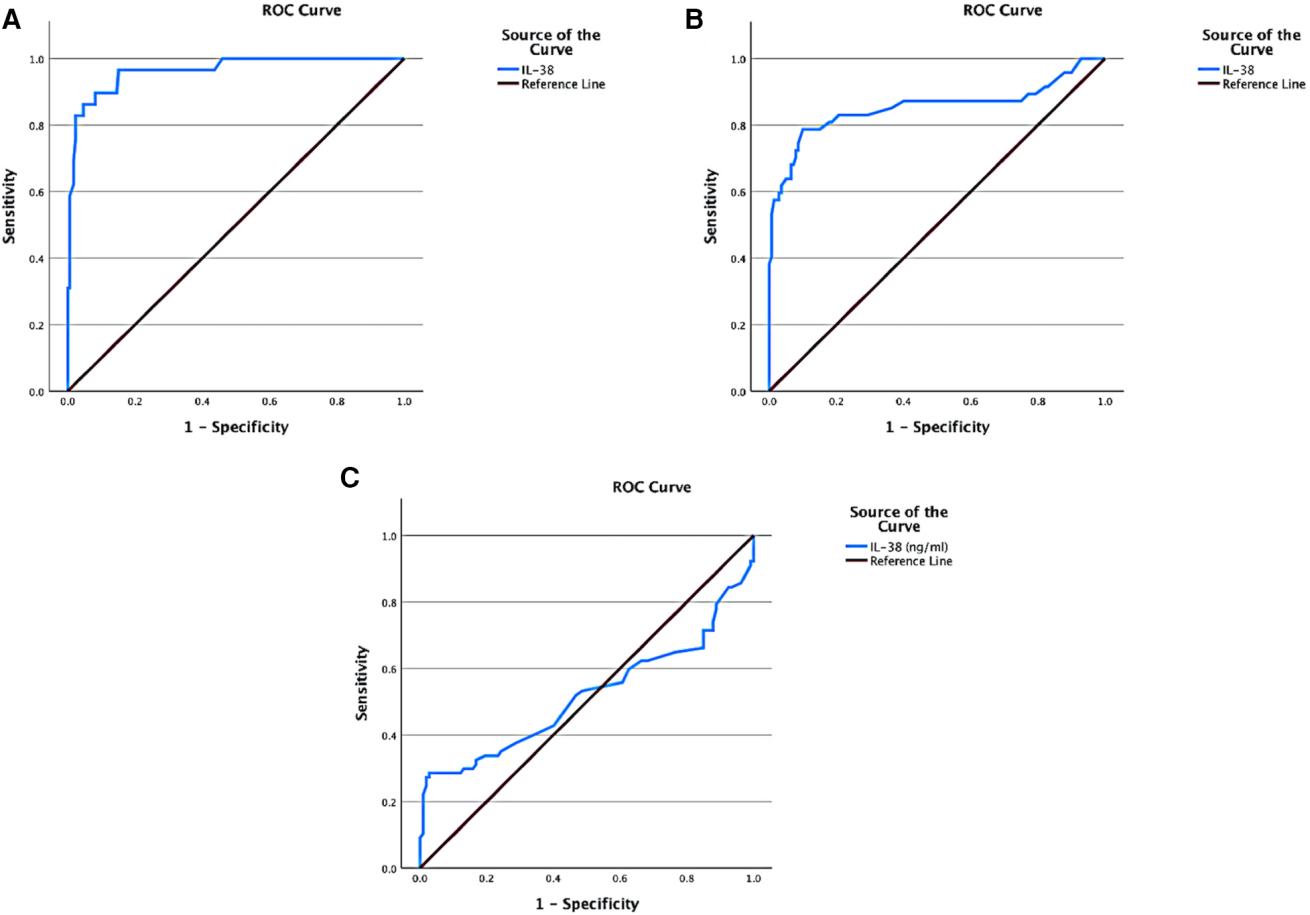
ROC analyses on the non-MACE occurrence predictive value of serum IL-38 level in patient subgroups. (**A**) ROC curve on IL-38 in patients with serum hs-CRP higher than 4.4 mg/L (AUC = 0.964). (**B**) ROC curve on IL-38 in patients with serum HbA1c higher than 6.2% (AUC = 0.853). (**C**) ROC curve on IL-38 in patients with smoking history (AUC = 0.533).

The area under the ROC curve (AUC) was used to verify the discrimination of the risk prediction model for non-MACE occurrence in the patients’ subgroups. The results showed that the AUC of non-MACE occurrence after PCI predicted by IL-38 levels in patients with high hs-CRP level was 0.964, and in patients with high serum HbA1c level was 0.853, suggesting that serum IL-38 level had good predictive efficacy in these patient subgroups (Figures 3A,B). In line with the stem-and-leaf plot mentioned above, AUC of non-MACE occurrence after PCI predicted by IL-38 levels in patients with smoking history was 0.517, indicating the lack of predictive power of IL-38 in these patients (Figure 3C).

## Discussion

CHD is a life-threatening cardiovascular disease with an incidence rate that increases over the years. Patients with CHD often combine multiple underlying diseases and metabolic disorders, including endothelial cell dysfunction, insulin resistance, blood hypercoagulation, and lipid metabolism disorders. These lead to multivessel lesions, poor collateral circulation, plaque instability, and distal small vessel disease, and further result in thrombosis and restenosis, the pathophysiological basis for coronary artery disease (13). With the application of vascular interventional treatment technologies such as PCI, the stenosis of blood vessels can be effectively relieved, but many patients still suffer from major cardiovascular adverse events after PCI, especially when there are environmental risks such as smoking and/or other disease complications (14, 15).

One of the biggest disease complications that lead to post-PCI MACE is diabetes. Abnormal blood glucose metabolism is an important factor associated with abnormal lipid metabolism in patients that can cause the accumulation of many risk factors for cardiovascular disease, such as hyperlipidaemia (16). Abnormal lipid metabolism secondary to insulin resistance is the key to the increase of TG in patients with CHD complicated with diabetes, and is closely related to the occurrence of diabetic macrovascular disease (17). HbA1c strongly correlates with the level of ambient glycaemia during a period of 2–3 months, so it reflects the usual daily fasting and post-prandial glucose levels as established by the American Diabetes Association (ADA) (18). In this study, logistic regression analysis demonstrated that serum HbA1c is an independent risk factor for MACE occurrence post PCI, which reconfirmed the need for blood glucose control in post-PCI care for patients with CHD, especially when they are complicated with diabetes.

Another pro-MACE complication is inflammation. Notably, hs-CRP is an important inflammatory marker for the occurrence of infection, inflammation, and injury in the body. It is involved in the formation of coronary atherosclerosis and is an independent risk factor for judging cardiovascular events (19). Elevated hs-CRP suggests an obvious inflammatory response in the intima of coronary arteries, which increases the instability of intravascular plaques and causes cardiovascular adverse events (20, 21). Here, as expected, logistic regression analysis demonstrated that serum hs-CRP is also an independent risk factor for MACE occurrence post PCI, indicating managing inflammation is one of the major needs for post-PCI care. Hence, anti-inflammatory pathways shall be considered potential therapeutic targets to reduce MACE occurrence.

As a newly discovered member of IL-1 family, interleukin-38 was found to bind the IL-36 receptor (IL-1R6) and exhibits anti-inflammatory properties. A relative deficiency of the B cell product IL-38 is associated with increased systemic inflammation in ageing, cardiovascular, and metabolic diseases and is consistent with IL-38 as an anti-inflammatory cytokine (11). There have been several reports on the link between IL-38 and hs-CRP. In this study, although serum levels did not appear different between the MACE and non-MACE groups shown by the univariate analysis in the overall cohort, IL-38 however was identified by the multivariate logistic regression test to have significant prediction power for post-PCI MACE, with its serum level reversely correlating with MACE occurrence. Taking into account its anti-inflammatory properties and association with HbA1c, we hypothesized that in these patients, a high level of IL-38 may function as a neutralizer to the MACE risk indicated by other risk factors such as hs-CRP and HbA1c. In fact, when focusing on the patient subgroups with abnormal hs-CRP or HbA1c serum levels, we did find a significant difference in serum IL-38 levels between MACE and non-MACE groups (as shown in Figure 2). Furthermore, the ROC analysis (Figure 3) indicated that in these patient subgroups, IL-38 alone is enough to predict non-MACE occurrence with a high AUC value (>0.8).

Another independent risk factor for post-PCI MACE evaluated by logistic regression test in the study is smoking history. The detrimental effect of smoking on the endothelial function and its impact on increasing cardiovascular risk has been widely recognized (22), which is in line with our findings. However, unlike in the subgroups with abnormal hs-CRP and HbA1c, IL-38 was not observed to significantly correlate with MACE risk in the smoking group, indicating a lack of similar direct interactions between the two. This could be explained by the fact that the mechanisms smoking induced are far more complicated than just inflammation, which IL-38 is believed to mainly target.

In all, a higher level of IL-38 in patients suggests that they have better ability to interact with the biochemical pathways involved in inflammation and/or metabolic disease complications, which are indicated by abnormal serum levels of hs-CRP and/or HbA1c. These findings will improve the understanding on biochemical pathways involved in post-PCI MACE and provide new insights for better post-procedure clinical management. However, further studies are needed to better understand the underlying molecular mechanisms. One limitation of the study is the lack of patients’ historic information on cardiovascular and metabolic disease comorbidities, as well as pharmacological therapies. These factors could be related to the occurrence of post-PCI MACE and should be studied in the future.

## Data Availability

The raw data supporting the conclusions of this article will be made available by the authors, without undue reservation.
